# Hidradenitis Suppurativa: A Systematic Review Integrating Inflammatory Pathways Into a Cohesive Pathogenic Model

**DOI:** 10.3389/fimmu.2018.02965

**Published:** 2018-12-14

**Authors:** Allard R. J. V. Vossen, Hessel H. van der Zee, Errol P. Prens

**Affiliations:** Department of Dermatology, Erasmus University Medical Center, Rotterdam, Netherlands

**Keywords:** acne inversa, pyoderma gangrenosum and acne, immune-mediated inflammatory disease, inflammatory bowel disease, auto-inflammation, nicastrin, PSTPIP1, obesity

## Abstract

**Background:** The pathogenesis of hidradenitis suppurativa (HS) is not fully understood. This systematic review examined the latest evidence for molecular inflammatory pathways involved in HS as a chronic inflammatory skin disease.

**Methods:** A systematic literature search was performed in PubMed/Medline and EMBASE from January 2013 through September 2017, according to the preferred reporting items for systematic reviews and meta-analyses (PRISMA). Findings on HS pathogenesis were also compared with those of other immune-mediated inflammatory diseases (IMIDs) in a non-systematic review. In addition, current therapeutic options for HS are briefly discussed on the basis of the findings for the inflammatory pathways involved in HS.

**Results:** A total of 32 eligible publications were identified by the systematic search; these were supplemented with three additional publications. The extracted data indicated that four key themes underlie the pathogenesis of HS and related syndromic conditions. First, nicastrin (*NCSTN*) and *PSTPIP1* mutations are directly associated with auto-inflammatory disease. Secondly, the up-regulation of several cytokines including tumor necrosis factor-α and T helper-17/interleukin-23 are connected to auto-inflammatory mechanisms in the pathogenesis of HS. Thirdly, the microbiome of lesional skin differs significantly vs. normal-appearing skin. Fourthly, HS risk is enhanced through physiological and environmental factors such as smoking, obesity, and mechanical friction. There is significant overlap between the pathogenesis of HS, its syndromic forms and other IMIDs, particularly with respect to aberrations in the innate immune response.

**Conclusions:** The evidence presented in this review supports HS as an auto-inflammatory skin disorder associated with alterations in the innate immune system. Based on these most recent data, an integrative viewpoint is presented on the pathogenesis of HS. Current management strategies on HS consist of anti-inflammatory therapies, surgical removal of chronic lesions, and lifestyle changes such as smoking cessation and weight loss. As large gaps remain in the understanding of the pathogenesis of HS, further research is warranted to ultimately improve the management and treatment of patients with HS and related syndromic conditions.

## Introduction

Hidradenitis suppurativa (HS) is a chronic, recurrent, inflammatory follicular occlusive disease, that usually presents after puberty with painful, inflamed lesions, predominantly at inverse body sites such as the axillae, inguinal and anogenital regions (1). The physiological and psychological consequences of HS can profoundly reduce a patient's quality of life (1, 2). Prevalence estimates in North America and Europe range from < 1 to 4% (3, 4).

The pathogenesis of HS is not fully understood. Current evidence highlights a complex multifactorial pathogenesis (5). A key triggering factor is the occlusion of the hair follicle, caused by keratosis and hyperplasia of the follicular epithelium leading to cyst development (6, 7). Subsequently, the cyst will rupture, causing a fierce immune response and inflammation that, depending on the severity, may progress to abscess and sinus tract development and scarring (6, 7). The name of the disease implies that sweating and bacterial infection are a fundamental part of the disease process. This is misleading and now considered a misnomer: no evidence has been found showing that HS is triggered by events in the apocrine or eccrine glands. Environmental risk factors reported to contribute to HS development include smoking and obesity (8). In addition, HS can occur with several co-morbid immune-mediated inflammatory diseases (IMIDs), notably inflammatory bowel disease (IBD) (9).

Clear evidence suggests the involvement of pro-inflammatory cytokines in immune dysregulation in HS, with elevated levels of tumor necrosis factor (TNF)-α, interleukin (IL)-1ß, IL-17 and interferon (IFN)-γ observed in HS lesions (5, 10, 11). Data also indicate the involvement of T helper (Th) cells, which accumulate in HS lesions, in the pathogenesis of HS (11, 12). In addition, studies have shown that antimicrobial peptides (AMPs) like cathelicidin (LL-37) and human β-defensin are increased in HS lesions compared with normal skin of HS patients (13). The use of TNF-α inhibitors such as adalimumab and infliximab have been associated with improvements in immune dysregulation in HS and support the importance of local molecular drivers in the pathogenesis of HS (1, 14, 15).

Furthermore, mutations in γ-secretase genes, whose gene products act on many substrates including Notch (16), suggest that Notch or other substrates of γ-secretase may play a role in the pathogenesis of HS. Interestingly, γ-secretase knock-out mice are characterized by a phenotype of multiple cutaneous cysts, a key feature of HS (17). To date it remains unclear whether the effects of Notch on follicle development or its immune role play a significant role in HS pathogenesis.

Rapidly evolving understanding in the auto-inflammatory arena is needed to improve awareness of HS, disease management, and ultimately improve patient outcomes. The aim of this systematic literature review was to summarize recent findings on the pathogenesis of HS and its syndromic forms, and to identify common pathways involved in HS pathogenesis and other IMIDs. Ultimately, we integrate the molecular pathways into a cohesive pathogenic model.

## Methods

A systematic review of recent original research was conducted according to the Preferred Reporting Items for Systematic review and Meta-Analyses Protocols (PRISMA-P) 2009 statement to identify the factors involved in the pathogenesis of HS (18).

A two-stage review process of PubMed/Medline and EMBASE databases was conducted using search strings designed to recognize studies reporting on auto-inflammatory disorders within the scope of HS (see Supplementary Tables 1, 2). The initial screening review identified studies published in English language from January 2007 through September 2017. After removing duplicate records, two reviewers independently screened the titles and abstracts in a double-blind manner and excluded those that did not meet the screening review inclusion criteria (Supplementary Table 3). Results were reviewed by a senior analyst for authentication and resolution of disagreements between the reviewers. Identified publications were reassessed according to the full-text review inclusion criteria; at this stage, the publication period was narrowed to January 2013 through September 2017 on the basis of the number of publications identified during screening; this was to ensure focus on the most recent data (Supplementary Table 3). Two reviewers, overseen by a senior analyst, independently assessed the remaining full-text copies of all relevant publications and any duplicate, low-quality or outdated publications were excluded. The bibliographies for all publications selected in the full-text review were also manually checked for relevant references.

Data were extracted from each relevant publication on study design (study setting, data source, study period), patient characteristics (sample size, mean age, sex, HS severity, disease location, notable comorbidities, smoking status), immunogenetic factors (genes, mutations or proteins involved), environmental factors (microbiological pathways, obesity, smoking, mechanical stress, sex, hormones), inflammatory pathway and cytokine status (pro-inflammatory, anti-inflammatory, proliferation, and growth factors).

In addition to the systematic review process, two informal literature searches were performed using the same databases, to facilitate discussion of the data. First, immunogenetic factors of HS are compared with other IMIDs such as Crohn's disease (CD), ulcerative colitis (UC), ankylosing spondylitis (AS), psoriasis and psoriatic arthritis (PsA), pyoderma gangrenosum (PG), and Behçet's disease. Second, actual therapeutic options for HS are briefly discussed on the basis of the inflammatory pathways involved in the pathogenesis of HS.

## Results

**Figure 1:**
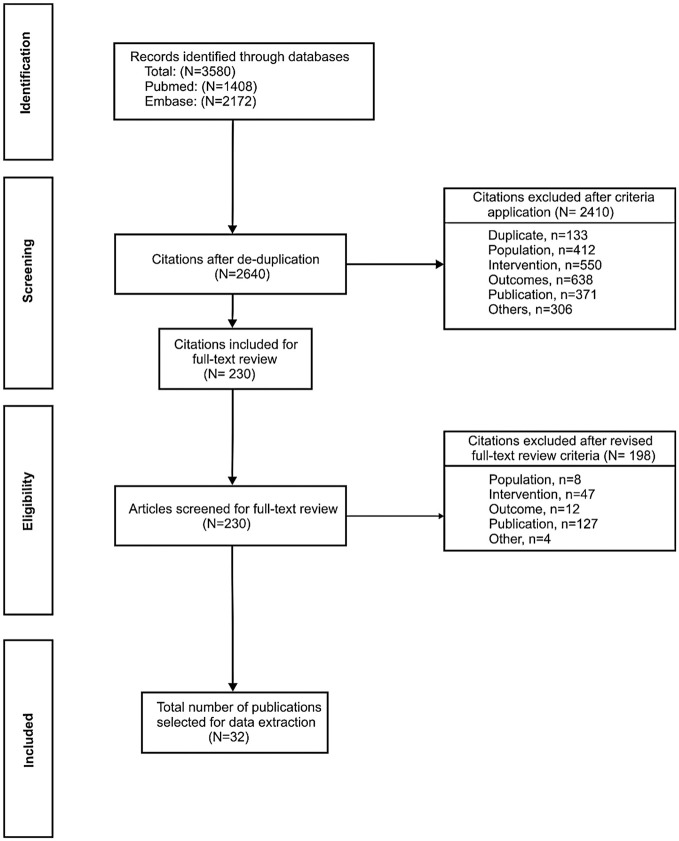
PRISMA flow diagram of included studies. To supplement the 32 publications identified through the systematic process, data from three additional publications were also reviewed as they were considered to provide relevant information; two had been excluded during the systematic search on the basis of their publication type (editorial/letter) (19, 20) and one was published subsequently to the search end date (21).

The initial search in PubMed and EMBASE identified a total of 3,580 records. Of these, 230 publications were screened for full-text review and 32 publications were selected for data extraction (Figure 1). Data from an additional three publications were also reviewed as they were considered to provide relevant information; two had been excluded during the systematic search on the basis of their publication type (editorial/letter) (19, 20) and one was published subsequently to the search end date (21).

The data extracted from these 35 publications were largely derived from four main lines of investigation in patients with HS and its syndromic forms: (1) genetic analyses (covered by 16 studies); (2) inflammatory marker levels (12 studies); (3) microbe analyses in lesions/expression of antimicrobial peptides (6 studies); and (4) contribution of physiological and environmental risk factors (11 studies) (Table 1).

**Table 1:** Characteristics of included studies.

**Factors associated with HS**	**Studies**		**Patients**	**Study location (n)**	**References**
	**Total, n**	**Per condition (n)**	**Per condition (n)**		
Genetics	16	HS/AI (8) PG (2) PAPA (2) PAC (1) PASH (2) PAPASH (1) SAPHO, RA, AS, SPA (1)	HS/AI (368) PG (15) PAPA/PAPA-like (~12)^α^ PAC (1) PASH (8) PAPASH (1) SAPHO (71), RA (125), AS (67), SPA (35)	Europe (7) Asia (5) Middle East (2) North America (2)	(3, 19, 20, 22–34)
Inflammatory markers	12	HS/AI (10) PG (2) PASH (1)	HS/AI (351) PG (15) PASH (7)	Europe (9) North America (2) Asia (1)	(5, 28, 31, 33, 35–42)
Microbiome	6	HS/AI (6)	HS (297)	Europe (6)	(43–48)
Physiological and environmental risk	11	HS/AI (11)	HS (738)	Europe (10) Asia (1)	(21, 26, 43, 45–52)

α *Marcos et al. (27) used donated cell cultures (n = 2). Note that three publications were included in addition to the 32 identified by the systematic review (see text for details) (19, 20, 52). AI, acne inversa; AS, ankylosing spondylitis; HS, hidradenitis suppurativa; PAPA, pyogenic arthritis, pyoderma gangrenosum, and acne; PAC, pyoderma gangrenosum, acne and ulcerative colitis; PASH, pyoderma gangrenosum, acne and suppurative hidradenitis; PG, pyoderma gangrenosum; RA, rheumatoid arthritis; SAPHO, synovitis, acne, pustulosis, hyperostosis, and osteitis; SPA, seronegative spondyloarthropathy*.

### Genetics

#### Loss of Function Mutations in γ-Secretase Complex Genes

Three studies identified by our review evaluated *NCSTN* gene mutations for the nicastrin protein subunit of γ-secretase in connection with HS pathogenesis (25, 26, 31). Genetic analyses in Chinese and Japanese families identified mutations in the *NCSTN* gene (c.647A>C, c.223G>A and c.582+1delG) carried by affected family members but not unaffected family members or healthy controls (25, 26). Meanwhile, an *in vitro* study in familial HS identified that mutations in *NCSTN* affect downstream signaling through Notch and/or phosphoinositide 3-kinase (PI3K) (31). However, NCSTN mutations in HS did not enhance cytokine production in LPS-stimulated peripheral blood mononuclear cells (20).

#### Mutations of Proline-Serine-Threonine Phosphatase Interacting Protein 1

Proline-serine-threonine phosphatase interacting protein 1 (PSTPIP1) is a cytoskeleton-associated adaptor protein, highly expressed in hemopoietic cells (29). The protein manifests its immunomodulatory effects through downregulation of CD2 (-triggered adhesion, regulation of c-Abl tyrosine kinase activity, and interaction with other immunity-related proteins including the Wiskott–Aldrich syndrome protein (WASp) (28) and pyrin, the familial Mediterranean fever (FMF) protein (29).

There is now evidence of mutations to the *PSTPIP1* gene in cases of pyoderma gangrenosum, acne and suppurative hidradenitis (PASH) and pyogenic arthritis, pyoderma gangrenosum, acne and suppurative hidradenitis (PAPASH) syndromes (19, 24). A p.E277D missense mutation was detected in the PASH case (24), whilst the patient with PAPASH had a heterozygous missense mutation (c.1213 C>T [p.Arg405Cys]) in exon 15 of *PSTPIP1*. Variations have also been reported in the *PSTPIP1* gene in other related syndromic conditions (22, 28, 29). First, a genetic analysis in a patient with a PAPA-like syndrome revealed a recessive inheritance pattern with a homozygous *PSTPIP1* mutation (c.773G>C and p.Gly258Ala), in contrast to a previously reported heterozygous polymorphism (22). Secondly, a patient with aggressive PG was found to have a novel *PSTPIP1-R405C* mutation (28). Data from this case study indicated that endogenous PSTPIP1 negatively regulates macrophage podosome formation and extracellular matrix degradation. Thirdly, a novel mutation in the *PSTPIP1* gene resulted in a case of pyoderma gangrenosum, acne and ulcerative colitis (PAC). The associated elevated IL-1β levels were responsive to the IL-1R antagonist anakinra (29). It is worth noting, however, that findings from a biochemical study suggested that *PSTPIP1* mutations associated with PAPA syndrome do not alter the negative regulatory role of PSTPIP1 in T-cell activation (27).

#### Other Genes Implicated in HS

In a study of 139 unrelated patients with HS, single nucleotide polymorphisms of the *IL-12Rb1* gene coding for the IL-12Rb1 receptor subunit did not genetically predispose to HS (23). However, their carriage was directly associated with the phenotype of HS, indicating the importance of the IL-12/IL-23 pathway for the pathogenesis of HS. Findings from a case-control study of two independent and genetically diverse cohorts of patients with HS from Greece (*n* = 163) and Germany (*n* = 98) suggested that the copy number of the β-defensin gene cluster (DEFB) both confers susceptibility for HS and modulates the disease phenotype (30).

In a study involving 298 Han Chinese patients with a range of auto-inflammatory diseases (Synovitis, Acne, Pustulosis, Hyperostosis and Osteitis [SAPHO], rheumatoid arthritis, AS and seronegative spondyloarthropathy), an AS-associated single-nucleotide polymorphism (rs6908425 in *CDKAL1*) was associated with the risk of developing SAPHO syndrome (32).

A genetic analysis of auto-inflammation in PG (13 patients) and the syndromic form PASH (7 patients) identified mutations in a range of auto-inflammatory genes (*MEFV, NLRP3, NLRP12, NOD2, LPIN2*, and *PSTPIP1*), suggesting the involvement of inflammatory pathways such as NLRP inflammasomes, cystolic pattern recognition sensors, the innate immune system, and IL-1β signaling (33).

In addition to the genetic analyses, two biochemical studies implicated other proteins in the pathogenesis of HS. Microarray data from one study suggested altered sphingolipid metabolism in HS skin lesions compared with normal skin (3).

In a study of surgically excised skin or skin punch biopsies, HS skin lesions showed on average 25-fold higher lipocalin 2 (LCN2) mRNA expression levels compared with the skin of healthy donors (34).

### Inflammatory Markers

There is increasing interest regarding the role markers of inflammation in patients with HS or other syndromic forms. A study in 14 patients with HS reported TNF-α-positive inflammatory cells in the dermis of patients but not in healthy controls (35). A study comparing the presence of different inflammatory cytokines in wound fluid specimens demonstrated elevated levels of IFN-γ and TNF-β in HS lesions compared with samples from age-matched chronic wound patients (5).

A retrospective study of HS outpatient medical files found a significant association between C-reactive protein (CRP) levels and neutrophil count with HS disease severity (36). A second study reported elevated serum CRP levels in patients with HS compared with healthy volunteers (38). A number of studies have reported elevated mRNA and/or protein levels of interleukins in the skin or serum. Alterations in the skin have been reported for IL-1β (33), CXCL-8/IL-8 (33, 37), IL-17/IL-17A (33), IL-32 (42), and IL-36/IL-36α/IL-36β/IL-36γ (37, 41). Alterations in the serum have been reported for IL-1β (38), IL-6 (38), CXCL-8/IL-8 (38), IL-10 (38), IL-12p70 (38), and IL-17/IL-17A (38, 39).

Keratinocytes isolated from non-lesional skin of patients with HS exhibited a pro-inflammatory profile in addition to an enhanced production of AMPs such as hBD-2, psoriasin (S100A7), and calgranulin (S100A8) (44), indicating that the skin immune system is already activated in the steady state.

### Microbiome

A number of studies investigated bacterial cultures from HS lesions and generated evidence implicating the involvement of microbes in disease pathogenesis. A histologic study of 42 patients with chronic HS identified bacterial aggregates (biofilms) in 67% of chronic lesion samples and in 75% of perilesional samples (47). The same author group conducted a case-control study of punch biopsy specimens and demonstrated that the microbiome in patients with HS differs significantly from that in healthy controls in both lesional and non-lesional skin (48). A microbial analysis of lesional vs. unaffected skin from 65 patients with HS identified anaerobic microbes in 83% lesions vs. 53% control samples, and the microbiome varied with disease severity (45). These bacteria were associated with low pathogenicity. An extensive prospective microbiological study identified two opportunistic bacterial pathogens associated with HS lesions (*S. lugdunensis* and anaerobic actinomycetes) (43). These pathogens can cause abscesses and severe infections. A cross-sectional study of 50 patients reported that bacterial colonization was correlated with severity and localization of HS lesions (46). Over two-thirds (68.8%) of patients with both aerobic and anaerobic bacteria had the most severe grade of HS (Hurley stage III).

### Physiological and Environmental Risk Factors

Findings from the literature review supported the involvement of previously suggested physiological and environmental risk factors, such as smoking and obesity, in HS (36, 49, 51). A postal follow-up survey study (*n* = 212) found the chance of remission from HS may be improved in non-smokers vs. smokers, and in non-obese (body mass index [BMI] < 30) vs. obese patients (49). In contrast, a retrospective study of inflammatory serum markers in HS outpatients found no association between smoking status and HS severity but smoking was associated with increased neutrophil counts (36). This study did find an association between increased BMI and HS severity, whereas there was no correlation between BMI and neutrophil counts.

Related to obesity, an analysis of 14 obese patients with HS described the role of mechanical stress (for example on the abdomen at the level of the waistband) in promoting the “Koebner phenomenon” in HS (51). The development of lesions at sites of traumatized but previously uninvolved skin highlights the importance of localized environmental factors in HS development. A hospital-based cross-sectional study conducted in the Netherlands reported a significantly higher average BMI in 106 patients with HS vs. 212 general dermatological patients (21). Among those patients identified as obese, bodyweight distribution was more peripheral in patients with HS than those without, consistent with enhanced friction due to overlapping skin folds.

Kromann and colleagues reported no clear effect of pregnancy or menopause on HS symptoms (49). However, in a cross-sectional survey based study, a substantial subset of women did experience HS-related alterations, with deterioration of HS around menses and amelioration of symptoms during pregnancy reported in 43% (*n* = 80) and 30% (*n* = 29) of the respondents, respectively (52).

### Evidence for Shared Pathology With Other IMIDs

To consider the above findings in relation to the pathogenesis of other established IMIDs, an informal literature review was conducted. Inflammatory bowel disease (CD and UC), AS, psoriasis, PsA, PG and Behçet's disease are characterized by different pathogeneses but they also share common immunological, genetic and risk factors (Table 2).

**Table 2:** Pathogenesis of established immune mediated inflammatory diseases in relation to hidradenitis suppurativa.

**Disease**	**Disease overview**	**Key^**α**^ genetic factor(s)**	**Key^**α**^ cytokine profile**	**Biologics**	**Risk factors**	**References**
HS	Inflammatory skin disease with genetic, immunological, and environmental background	γ-secretase (NCSTN), PSTPIP1	Th1, Th17 IL-1β, 6, CXCL/IL-8, 12, 17, 23, IFN-γ, TNF-α	Anti-TNF-α inhibitors	Smoking, obesity, mechanical friction	(5, 19, 24–26, 31, 33, 36–38, 41, 49, 51)
**IBD**						
CD	Imbalance between gut microbiome and host immune system with genetic background	NOD2 (CARD15), ATG16L1, IRGM, FUT2, OCTN, TNFSF15, IL10, IL12B, IL23R, HLA, STAT3, JAK2, TNFSF15, MUC1	Th1, Th17 IL-1β, 6, 12, 17, 23, IFN-γ, TNF-α	Anti-TNF-α inhibitors	Smoking, diet, vitamin D deficiency, medications, enteric infections	(53–55)
UC	Imbalance between gut microbiome and host immune system with genetic background	HNF4A, CDH1, LAMB1, GNA12, SLC9A, TNFSF14, ECM1, IL10, IL12B, IL23R, HLA, STAT3, JAK2, TNFSF15, MUC1	Th2, Th17 IL-1β, 6, 12, 13, 17, 23 TNF-α	Anti-TNF-α inhibitors	Non-smoking, appendectomy, diet, vitamin D deficiency, medications, enteric infections	(55)
AS	Imbalance between gut microbiome and host immune system with genetic background	HLA-B27, HLA-B40, ERAP1/2, CARD9, IL12B. IL23R, IL27, STAT3, JAK2, TYK2	Th17 IL-6, 17, 22, 23, 26, IFN-γ, TNF-α	Anti-TNF-α inhibitors	Infection, smoking, testosterone	(56–62)
Psoriasis	Inflammatory skin disease with genetic and immunological background	PSOR1, HLA-C, ERAP1, LCE3D, IL12B, IL23R, TNFAIP3, ZNF313, TYK2,	Th1, Th17 IL-2, 17, 22, 23, 26, TNF-α, IFN–γ	Anti-TNF-α inhibitors, T cell targeted therapies	Obesity, infection	(63–68)
PsA	Inflammatory arthritis associated with psoriasis with genetic, immunological, and environmental background	HLA-B. HLA-C, OCTN IL12B, IL23R	Th1, Th17 IL17, 23, TNF-α	Anti-TNF-α inhibitors	Physical trauma, smoking, obesity, infection, heredity	
PG	Inflammatory, ulcerating, neutrophilic skin disease with genetic, immunological, and environmental background	MEFV, NLRP3, NLRP12, NOD2, LPIN2, PSTPIP1	IL-1β, 17, TNF-α	Anti-TNF-α inhibitors	Physical trauma, non-smoking, metabolic syndrome	(33, 69–71)
Behçet's disease	Multi-systemic, inflammatory, vasculitis with genetic, immunological, and environmental background	HLA-B5, ERAP1 IL10, IL12RB2, IL-23R,STAT4, CCR1-CCR3, KLRC4, TNFAIP3, FUT2	Th1, Th17 IL-6, 11, 17, 21, 22, 26, TNF-α, Chitinase3-like1, gp130/sIL−6Rb, sTNF-R1, sTNF-R2	Anti-TNF-α inhibitors, anti-IL1, INF-α	Non-smoking, obesity, infection	(69, 72–74)

α *Data summarize key genes and cytokines involved in the pathogenesis of these diseases but many other genes, cells types and mediators are involved. AS, ankylosing spondylitis; CD, Crohn's disease; HS, hidradenitis suppurativa; PG, pyoderma gangrenosum; PsA, psoriasis and psoriatic arthritis; UC, ulcerative colitis*.

Several cytokines are systemically-raised in many of these IMIDs, particularly those implicated in the Th1 and Th17 responses, including TNF-α, IL-12/23, IL-17, IL-12, IFN-γ, IL-1β, and the IL-1 family including IL-36 (33, 37, 60, 67, 73). Several of the inflammatory cytokines have also been shown to be upregulated in HS [e.g., IFN-γ (5), IL-2, TNF-α (33, 35) and TNF-β (5)] are produced by Th1 cells, implicating the Th1 response in the pathogenesis of HS. Furthermore, the IL-36 family, also found to be upregulated in HS (37, 41), plays an important role in the modulation of Th1 and Th17 immune responses.

Data also support the notion of shared genetic pathways of inflammation. For example, *NOD2* mutations in CD are identified in PG and PASH (33) but conflicting evidence concerns their association with psoriasis and PsA (63, 75). *MEFV* mutations are found in FMF as well as PG and PASH; co-occurrence of FMF and HS is not uncommon (33, 76). Other genes conferring susceptibility in CD, such as *OCTN*, are associated with PsA (63).

In addition to shared genetic factors, the overlap in risk factors observed for different IMIDs also highlights the similar mechanisms that account for them. For example, smoking may confer a protective role in the pathogenesis of UC, PG and Behçet's disease but increases susceptibility in CD, AK, psoriasis and PsA (Table 2). With the exception of AK and conflicting evidence for Behçet's disease (56, 74), the pathogenesis of most IMIDs appears unrelated to sex-specific factors. Understanding the distinct and shared genetic, immunologic and risk factor profiles of IMIDs will aid the development of effective treatments to target the pathogenic mechanisms involved and modify the disease course.

It has previously been proposed that the link between HS and other conditions with demonstrated systemic pathology may be attributed to common genetic or environmental factors and/or shared inflammatory pathways (77).The data identified in this review demonstrate the significant overlap between the pathogenesis of HS and the aforementioned IMIDs. The most striking similarity among these diseases is that of aberrations in the innate immune response, particularly the IL-23/Th17 pathway (Table 2).

### Integrated Viewpoint on HS Pathogenesis “Sequence of Events”

By identifying the latest publications on the pathogenesis of HS and evaluating it in the context of more established pathogenic mechanisms for known IMIDs, this review has collated substantial evidence that HS is a chronic immune-mediated auto-inflammatory disease with a multifactorial pathogenesis.

Four key themes have emerged from this review. First, genetic factors play a key role in causing HS. Mutations in a range of genes, including *NCSTN* mutations in the γ-secretase complex and *PSTPIP1* mutations, are directly associated with auto-inflammatory disease (26, 27, 29, 31). However, the majority of HS cases appear to be non-familial, suggesting the existence of separate subsets and the need for stratification within patients diagnosed with HS (25). Secondly, the up-regulation of cytokines including TNF-α and a range of cytokines (predominantly Th17-related) are connected to auto-inflammatory mechanisms in the pathogenesis of HS (5, 35, 38). Thirdly, there is an alteration in the local microbiome of normal-appearing vs. lesional skin (43, 45, 47, 48). Data also suggest that bacterial aggregates are associated with inflammation of chronic HS lesions, and it is proposed that they most likely occur as a secondary event, possibly due to predisposing local anatomical changes such as sinus tracts (tunnels), keratinous detritus and dilated hair follicles (47). Finally, enhancement of HS risk occurs via a range of physiological and environmental factors such as smoking, obesity and mechanical friction (21, 36, 49, 51).

**Figure 2:**
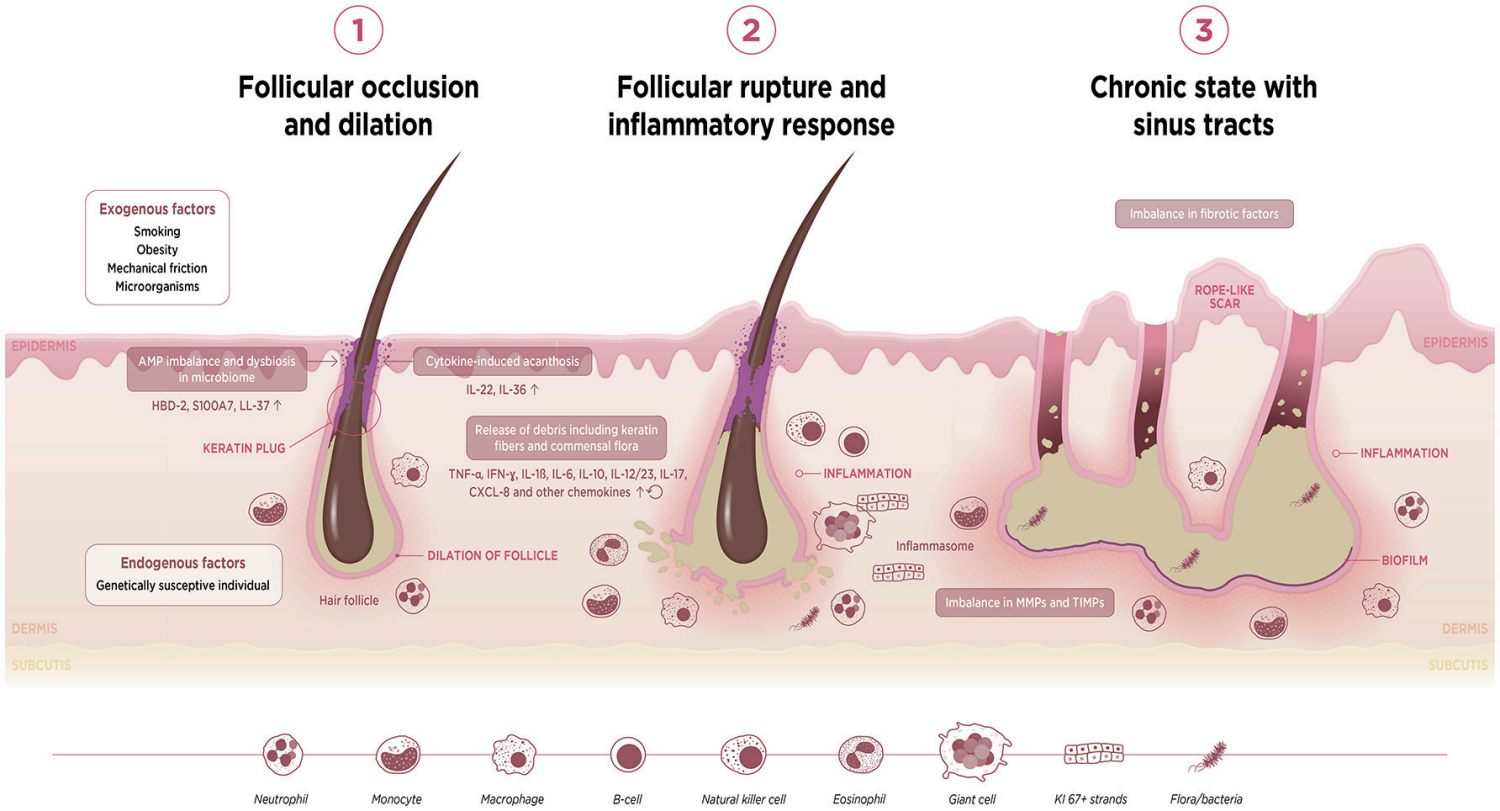
Schematic diagram to illustrate postulated sequence of events underlying HS pathophysiology. AMP, antimicrobial protein; HBD, human beta-defensin; IFN, interferon; IL, interleukin; MMP, matrix metalloproteinase; TIMP, tissue inhibitor of metalloproteinase; TNF, tumor necrosis factor.

On the basis of the evidence reviewed here, we are able to take a cohesive view and to propose a three-stage sequence of events that contribute to the pathogenesis of HS. This integrated viewpoint is illustrated schematically in Figure 2.

The first event is follicular occlusion with subsequent dilation. This may be driven by endogenous factors in individuals harboring a genetic predisposition for an enhanced risk of infundibular keratinisation and cyst formation. Exogenous factors such as smoking, mechanical friction and metabolic changes such as obesity—which is associated with acanthosis—also contribute to occlusion of the follicular isthmus. Furthermore, occlusion of the hair follicle may lead to a dysregulation of the homeostatic keratinocyte symbiosis and microbial dysbiosis, making the skin prone to a Th1/Th17-driven inflammatory disease.

**Figure 3:**
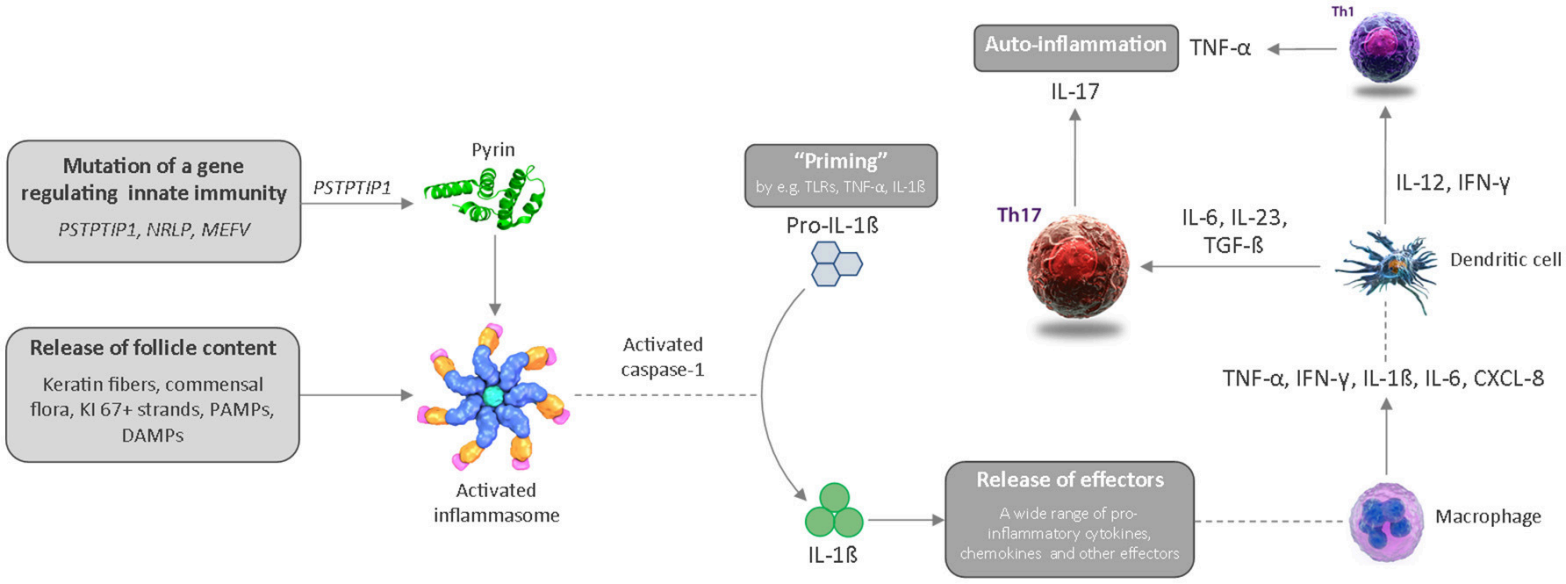
Schematic diagram illustrating activation of the inflammasome in HS and related syndromes, which ultimately results in an auto-inflammatory immune response. CXCL, chemokine ligand; DAMP, danger-associated molecular pattern; IL, interleukin; IFN, interferon; PAMP, pathogen-associated molecular pattern; Th, T helper cell; TLR, toll-like receptor; TNF, tumor necrosis factor.

The second event is rupture of the dilated follicle. The scattering of follicle content in the dermis including keratin fibers, commensal flora or pathogen- and damage-associated molecular patterns (PAMPs/DAMPs) triggers an acute and severe immune response. The anatomical location, i.e., the inverse body areas, and enhanced mechanical friction at these predilection sites facilitates the inward rupture and extension of inflammation. We argue that release of the follicular debris into the dermis results in simultaneous activation of multiple inflammatory pathways, particularly Th17/IL-23, the (NLRP) inflammasomes and innate receptors (toll-like receptors, TLRs such as TLR2). Activation of the inflammasome in HS and related syndromes including PASH and PAPA(SH) is illustrated schematically in Figure 3. This is accompanied by histological alterations with a diverse cell infiltrate characterized by the mixed participation of monocytes, neutrophils, multinucleated giant cells, B-cells, plasma cells, T-cells, and natural killer cells, leading to an erythematous nodule or fluctuating abscess.

The third event is chronic inflammation with sinus tract or tunnel formation. Following follicular rupture, sequestered proliferating Ki-67+ epithelial strands promote continuous activation of the immune system. The presence of epithelial strands in the dermis, in addition to an imbalance in matrix metalloproteinases (MMPs) and tissue inhibitors of metalloproteinase (TIMPs), and increased activity of fibrotic factors such as tissue growth factor (TGF)-ß 1-2-3, may lead to scarring and the development of sinuses/tunnels or fistulae, a hallmark of chronic HS. These intracutaneous (partly) epithelialized cavities provide an excellent habitat for biofilm-producing bacteria, which are able to continuously trigger inflammation with associated purulent drainage. Furthermore, we hypothesize that circulating pro-inflammatory cytokines and chemokines from chronic lesions may activate the immune system of the hair follicle in distant predilection sites.

### Current Therapeutic Options for HS

HS management usually consists of the combination of both medical therapies and surgical interventions. The main treatment goal, to improve patients' quality of life, can be achieved by reducing the inflammation-related pain and purulent discharge, limiting the incidence and duration of flares, and removing chronic lesions using surgical techniques (78). A short overview of current treatment options including the therapeutic target and/or the suggested pathophysiological link(s) is depicted in Table 3. These data summarize anti-inflammatory therapies in addition to surgery and lifestyle changes such as smoking cessation and weight loss. First-line treatment options include the use of antibiotics with anti-inflammatory properties, e.g., the tetracyclins and the combination of clindamycin and rifampicin (80, 87). The anti-TNF-α agents adalimumab and infliximab should be considered, respectively, as first- and second-choice biologics for moderate-to-severe HS after failure of systemic antibiotics (14, 90). Ustekinumab (anti-IL-12/23p40) is potentially effective in the treatment of HS (89), whereas the results of two randomized controlled trials investigating IL-17 antagonists are awaited (ClinicalTrials.gov Identifiers NCT02421172 and NCT03248531). Other promising treatment options are MABp1, targeting IL-1α for HS patients not eligible for adalimumab, and apremilast for patients with moderate HS (92, 94).

**Table 3:** Short overview of actual treatment options for hidradenitis suppurativa, based on Van Straalen et al. (78).

**Treatment options^**α**^**	**Therapeutic target or suggested pathophysiological link**	**References^**ß**^**
**LIFESTYLE CHANGES**		
Smoking cessation	Reduction of follicular acanthosis; less xenobiotic metabolism, e.g., via the aryl hydrocarbon receptor, with potential restoration of alterations in the immune response	(8, 49)
Weight loss	Improvement of the metabolic state, thereby reducing follicular acanthosis; less mechanical friction as a result of less overlapping skin folds with potential restoration of the local microbiome	(79)
**LOCALLY ADMINISTERED AGENTS**		
Clindamycin 1% lotion	Anti-inflammatory and antibacterial properties; for acute lesions	(80)
Resorcinol 15% cream	Removal of follicular plugging (prophylactic effect) and early rupture of an abscess due to its keratolytic properties; antiseptic properties	(81, 82)
Intralesional triamcinolone	Pan-cell inhibitor; for acute lesions to eradicate the inflammatory cell infiltrate	(83)
**SYSTEMIC ANTIBIOTICS**		
Tetracyclins; clindamycin and rifampicin; moxifloxacin, rifampicin, and metronidazole	Various modulations in the immune response, e.g., inhibition of neutrophilic migration and chemotaxis, inhibiting IL-1ß and TNF-α secretion, upregulation of IL-10, inhibition of the angiogenesis, and suppressing T-cell function; antibacterial effects	(80, 84–88)
**BIOLOGICS**		
Adalimumab, infliximab; ustekinumab; anakinra; MABp1	Monoclonal antibodies targeting TNF-α, IL-12/23p40, IL-1R, and IL-1α, respectively	(14, 89–92)
**SMALL MOLECULE DRUGS**		
Apremilast	Inhibits PDE-4 in various inflammatory cell types, thereby modulating several pro- and anti-inflammatory cytokines	(93, 94)
**SURGERY**		
Deroofing, excision	Removal of irreversibly damaged skin, i.e., sinus tracts or nodules/cysts recurring on fixed locations	(95, 96)

α *Data summarize the most important medical and surgical therapeutic options in addition to lifestyle changes. The majority of the remaining evidence to guide management decisions is based on case reports, case series with fewer than 10 patients, small cohort studies, and expert opinion, which are all not included in this overview. Based on highest level of evidence or largest cohort for each intervention. IL, interleukin; PDE-4, phosphodiesterase-4; TNF, tumor necrosis factor*.

## Limitations

This review was subject to certain limitations. PubMed/Medline and EMBASE were the only two databases used to identify eligible studies. Any studies published in journals not listed in PubMed/Medline and EMBASE are omitted from this review. The extent of recent published evidence relating to the pathogenesis of HS and related syndromic conditions is limited. Finally, the review of other IMIDs for comparison with HS was not systematic, and conclusions drawn from this informal review must be interpreted with this methodology in mind.

## Future Research

Large gaps still remain in the understanding of the pathogenesis of HS. Therefore, further research is warranted to ultimately improve the management and treatment of patients with this disease. Genetic research should aim to add more detail to the proposed mechanism by which loss of function of NCSTN or of other γ-secretase proteins causes familial HS and to better stratify patients with HS. Immunologic studies should focus on molecular drivers of tissue inflammation and injury in HS and the relationship between HS cytokine profile and disease activity. Microbiome research is needed to better characterize the disruption to the microbial ecosystem and to elucidate whether the disruption causes the disease or whether the disease causes the dysbiosis. High-throughput metagenomic methods can make this work possible. Finally, it will be important to focus research on the interaction of environmental factors and immunogenetic factors.

## Author Contributions

All authors contributed equally to the design of the literature search, the analysis of results and development of the paper.

### Conflict of Interest Statement

HvdZ: advisory board member for AbbVie, InflaRX, and Galderma. EP: consultant, speaker, principal investigator or received grants from: AbbVie, Amgen, Biogen, Celgene, Eli Lilly, Janssen-Cilag, Novartis, Pfizer, and UCB. The remaining author declares that the research was conducted in the absence of any commercial or financial relationships that could be construed as a potential conflict of interest.
