# Supplementary material for: Hidradenitis Suppurativa: A Systematic Review Integrating Inflammatory Pathways Into a Cohesive Pathogenic Model
Source: Front Immunol. 2018 Dec 14;9:2965. doi: 10.3389/fimmu.2018.02965 (PMC6302105; doi:10.3389/fimmu.2018.02965)
Supplement: Supplementary file 1 [file Table_1.DOCX]

**Supplementary Material**

**Supplementary Table 1: Search in EMBASE on September 21, 2017**

| Search # | Query | Records |
| --- | --- | --- |
| 1. | 'suppurative hidradenitis'/exp OR 'suppurative hidradenitis' | 2436 |
| 2. | 'hidradenitis suppurativa':ab,ti OR 'acne inverse':ab,ti OR 'acne-inverse':ab,ti OR 'acne-inversa':ab,ti OR 'acne inversa':ab,ti OR 'hidradenitis axillaris':ab,ti OR 'sweat gland abscess':ab,ti OR 'apocrine acne':ab,ti OR 'apocrinitis':ab,ti OR 'pyodermia fistulans significa':ab,ti OR 'acne conglobata':ab,ti | 2495 |
| 3. | ('suppurative hidradenitis'/exp OR 'suppurative hidradenitis') OR 'hidradenitis suppurativa':ab,ti OR 'acne inverse':ab,ti OR 'acne-inverse':ab,ti OR 'acne-inversa':ab,ti OR 'acne inversa':ab,ti OR 'hidradenitis axillaris':ab,ti OR 'sweat gland abscess':ab,ti OR 'apocrine acne':ab,ti OR 'apocrinitis':ab,ti OR 'pyodermia fistulans significa':ab,ti OR 'acne conglobata':ab,ti | 3060 |
| 4. | ('verneuil':ab,ti OR 'velpeau':ab,ti OR 'foxden disease':ab,ti) AND 'hidradenitis suppurativa':ab,ti | 55 |
| 5. | 'papa':ab,ti OR 'papa syndrome':ab,ti OR 'pyogenic arthritis, pyoderma gangrenosum and acne':ab,ti OR 'pash':ab,ti OR 'pyoderma gangrenosum, acne and suppurative hidradenitis':ab,ti OR 'papash':ab,ti OR 'pa-pash':ab,ti OR 'pyogenic arthritis, acne, pyoderma gangrenosum and suppurative hidradenitis':ab,ti OR 'sapho':ab,ti OR 'synovitis, acne, pustulosis palmoplantaris, hyperostosis, osteitis':ab,ti OR 'synovitis, acne, pustulosis, hyperostosis, and osteitis':ab,ti OR 'pyoderma gangrenosum, acne, and ulcerative colitis':ab,ti OR 'psapash':ab,ti OR 'psa-pash':ab,ti OR 'pyoderma gangrenosum, acne, suppurative hidradenitis, and axial spondyloarthritis':ab,ti OR 'pyoderma gangrenosum, acne vulgaris, suppurative hidradenitis, and axial spondyloarthritis':ab,ti OR 'pyoderma gangrenosum, acne conglobata, suppurative hidradenitis, and axial spondyloarthritis':ab,ti |  |
| 6. | 'pyoderma gangrenosum':ab,ti OR 'pyogenic arthritis':ab,ti OR acne:ab,ti OR syndrome:ab,ti OR hidradenitis:ab,ti | 1054877 |
| 7. | #5 AND #6 | 955 |
| 8. | (PAC:ab,ti OR PASS:ab,ti) AND 'pyoderma gangrenosum':ab,ti | 7 |
| 9. | #1 OR #2 OR #3 OR #4 OR #7 OR #8 | 3914 |
| 10. | 'immuno-genetics':ab,ti OR 'immunogenetics':ab,ti OR gene*:ab,ti OR environ*:ab,ti OR lifestyle:ab,ti OR 'life style':ab,ti OR factor:ab,ti OR predict*:ab,ti OR progn*:ab,ti OR muta*:ab,ti OR 'gamma secretase':ab,ti OR 'gammasecretase':ab,ti OR 'γ-secretase':ab,ti OR 'γ secretase':ab,ti OR notch:ab,ti OR 'patho-genesis':ab,ti OR pathogenesis:ab,ti OR pathophysiology:ab,ti OR 'patho-physiology':ab,ti OR 'disease development':ab,ti OR 'development of disease':ab,ti OR etio*:ab,ti OR aetio*:ab,ti OR modul*:ab,ti OR express*:ab,ti OR pathway:ab,ti OR mech*:ab,ti OR 'gene expression':ab,ti OR 'rna expression':ab,ti OR 'sequencing':ab,ti OR 'cause':ab,ti OR caus*:ab,ti OR immun*:ab,ti OR patho*:ab,ti OR 'disease initiation':ab,ti OR sever*:ab,ti | 15090935 |
| 11. | #9 AND #10 | 2294 |
| 12. | #10 AND [1987-2017]/py | 2172 |

**Supplementary Table 2: Search in PubMed on September 21, 2017**

| Search # | Query | Records |
| --- | --- | --- |
| #1 | Search ("hidradenitis suppurativa"[MeSH Terms] OR "hidradenitis suppurativa"[tiab] OR acne invers*[tiab] OR "hidradenitis axillaris"[tiab] OR "sweat gland abscess"[tiab] OR "Apocrine acne"[tiab] OR "Apocrinitis"[tiab] OR Pyodermia fistulans significa[tiab] OR "Foxden disease"[tiab] OR "Acne conglobata"[tiab] OR "hidradenitis"[tiab]) | 2199 |
| #2 | Search ("Verneuil"[tiab] OR "Velpeau"[tiab]) | 64 |
| #3 | Search "hidradenitis"[tiab] | 1800 |
| #4 | Search (#2 AND #3) | 11 |
| #5 | Search ("Pyoderma Gangrenosum"[tiab] OR "pyogenic arthritis"[tiab] or "Acne" OR "syndrome"[Tiab] OR "hidradenitis"[tiab]) | 808874 |
| #6 | Search (PAPA OR "PAPA syndrome" OR "pyogenic arthritis, Pyoderma Gangrenosum and acne" OR "PASH" OR "Pyoderma Gangrenosum, acne and suppurative hidradenitis" OR "PA-PASH" OR "PAPASH" OR "pyogenic arthritis, acne, Pyoderma Gangrenosum and suppurative hidradenitis" OR "SAPHO" OR "synovitis, acne, pustulosis palmoplantaris, hyperostosis, osteitis" OR "synovitis-acne-pustulosis-hyperostosis-osteitis" OR "Pyoderma gangrenosum, acne and ulcerative colitis" OR "pyoderma gangrenosum, acne vulgaris, hidradenitis suppurativa and ankylosing spondylitis" or "pyoderma gangrenosum, acne vulgaris, suppurativa hidradenitis and ankylosing spondylitis" OR "pyoderma gangrenosum, acne, hidradenitis suppurativa and ankylosing spondylitis" or "pyoderma gangrenosum, acne, suppurativa hidradenitis and ankylosing spondylitis" OR "pyoderma gangrenosum, acne conglobata, hidradenitis suppurativa and ankylosing spondylitis" or "pyoderma gangrenosum, acne conglobata, suppurativa hidradenitis, and ankylosing spondylitis" OR "PsAPASH" OR "PsA-PASH" OR "psoriatic arthritis, acne, Pyoderma Gangrenosum and suppurative hidradenitis") | 6256 |
| #7 | Search (#5 AND #6) | 968 |
| #8 | Search ("PASS"[tiab] OR "PAC"[tiab]) | 51838 |
| #9 | Search "Pyoderma Gangrenosum"[tiab] | 2611 |
| #10 | Search (#8 AND #9) | 4 |
| #11 | Search (#1 OR #4 OR #7 OR #10) | 3085 |
| #12 | Search ("Immunogenetics"[tiab] OR Immuno*[tiab] OR"immuno-genetics"[tiab] OR "immunogenetics"[tiab] OR gene*[tiab] OR environ*[tiab] OR "lifestyle"[tiab] OR "life-style"[tiab] OR factor[tiab] or predict*[tiab] OR progn*[tiab] OR risk factor*[tiab] OR muta*[tiab] OR "gamma secretase"[tiab] OR "gammasecretase"[tiab] OR "Î³-secretase"[tiab] OR "Î³ secretase"[tiab] OR notch[tiab] OR pathogenesis[tiab] OR "patho-genesis"[tiab] OR patho*[tiab] OR "pathogenesis"[tiab] OR "pathology"[tiab] OR "pathophysiology"[tiab] OR "patho-physiology"[tiab] OR "disease development"[tiab] OR "development of disease"[tiab] OR etio*[tiab] OR aetio*[tiab] OR modul*[tiab] OR express*[tiab] OR pathway[tiab] OR mech*[tiab] OR "gene expression"[tiab] OR "RNA expression"[tiab] OR "sequencing"[tiab] OR caus*[tiab] OR "disease initiation"[tiab] OR sever*[tiab]) | 10236807 |
| #13 | Search (#11 AND #12) | 1474 |
| #14 | Search (#11 AND #12) Filters: Publication date from 1987/01/01 to 2017/09/21 Sort by: Publication Date | 1408 |

**Supplementary Table 3** Eligibility Criteria for Screening and Full-text Review

|  | **Inclusion** | | **Exclusion** | |
| --- | --- | --- | --- | --- |
|  | **Screening** | **Full-text** | **Screening** | **Full-text** |
| **Population** | Patients with HS, acne inversa, hidradenitis axillaris, sweat gland abscess, apocrine apocrinitis, pyodermia fistulans significa, acne conglobata, verneuil's disease, velpeau’s disease, foxden disease, syndromic forms of HS including PASH, PAPA, PA-PASH, SAPHO, PsA-PASH | | Patients without HS | |
|  | *Animal model studies* | *Patients with IMIDs: CD, UC, PG, AS, PsA, and Behcet's disease* |  | *Patients with NEH, acne vulgaris, Dowling-Degos syndrome, Cushing’s syndrome, palmoplantar hidradenitis patients Report HS as a secondary indication Patients undergoing current treatment Animal studies* |
| **Interventions** | N/A | | Publication focused on drug treatments | |
|  |  |  |  | *Publication focused on drug induced HS/IMID* |
| **Outcomes** | Factors involved in pathogenesis and severity of HS and its syndromic forms such as immunogenetic factors, environmental factors, inflammatory pathways of innate and adaptive immunity, genetics  Cross-over with other IMIDs | | Not reporting pathogenesis and related factors for HS and its syndromic forms  Describing clinical features of HS patients  Describing natural history of HS patients  Focused on quality of life of HS patients  Purely diagnostic studies | |
|  |  |  |  | *Reporting only bacterial presence in cultures from HS patients (non-pathogenic relationships)* |
| **Design** | Cohort studies, observational studies, case reviews/reports and basic science publications | | Systematic reviews, meta-analyses, narrative reviews, editorial Meeting or conference abstracts or posters | |

Italic text indicates differences in screening and full text review inclusion criteria

AS, ankylosing spondylitis; CD, Crohn’s disease; IMID, immune mediated inflammatory diseases; NEH, neutrophilic eccrine hidradenitis; PA, Pyogenic arthritis; PAPA, pyogenic arthritis, pyoderma gangrenosum and acne; PASH, pyoderma gangrenosum, acne, and hidradenitis suppurativa; PG, pyoderma gangrenosum; PsA, psoriasis and psoriatic arthritis; SAPHO, synovitis, acne, pustulosis, pyogenic arthritis, hyperostosis, and osteitis; UC, ulcerative colitis
